# Categorisation of myopia progression by change in refractive error and axial elongation and their impact on benefit of myopia control using orthokeratology

**DOI:** 10.1371/journal.pone.0243416

**Published:** 2020-12-29

**Authors:** Pauline Cho, Sin Wan Cheung, Maureen V. Boost

**Affiliations:** School of Optometry, The Hong Kong Polytechnic University, Kowloon, Hong Kong SAR, China; Universita degli Studi di Firenze, ITALY

## Abstract

**Aims:**

To compare the value of pre-treatment axial elongation (AE) and changes in refractive sphere (M change) for predicting the success in orthokeratology (ortho-k), in order to better identify suitable candidates for myopia control.

**Methods:**

This study further analysed the data of 66 subjects receiving 7-month ortho-k treatment, following a 7-month observation period, during which single-vision spectacles were worn. Rate of myopia progression was determined by AE and M change and subjects categorised as slow, moderate, or rapid progressors based on these changes. Outcomes of myopia control, based on the AE reduction after ortho-k, were classified as ‘ineffectual’, ‘clinically insignificant’, or ‘beneficial’.

**Results:**

Of the 20 subjects, initially categorised as slow by AE and, of whom 95% were similarly categorised by M change, none benefitted from ortho-k. In contrast, of the 22 subjects with moderate AE, 77% and 23% displaying slow and moderate M change, respectively, the majority (73%) benefitted from ortho-k lens wear. The 24 subjects with rapid AE were poorly identified by M change, with only 21% correctly categorised. The vast majority of rapid progressors showed significant benefit after ortho-k.

**Conclusion:**

Progression of AE is a good indicator of subsequent success of ortho-k treatment. Delaying commencement of therapy is prudent for children with slow progression as results indicate that they would be unlikely to benefit from this intervention. As change in refractive error frequently underestimates rapid progression of AE, its value for identifying appropriate candidates for myopia control is poor.

## Introduction

The dramatic worldwide increase in myopia has led to increasing concerns about the long term consequences of this condition, especially, in patients progressing to high myopia [1–3]. In most cases, myopia development commences in early childhood and progresses until late adolescence [4–6]. The surge in interest in myopia has resulted in development and prescription of various interventions for its control in children. These include orthokeratology (ortho-k) [7–9], atropine instillation [10, 11], specially designed soft contact lenses [12–14] and DIMS spectacles [15, 16]. Of these, ortho-k has been well received and is popular with patients and their parents, especially in East Asia, where prevalence of myopia is particularly high [1, 2]. Use of low concentration atropine, which requires medical prescription, has also gained high popularity as it does not require overnight lens wear or stringent lens care routines. This is particularly the case in regions where optometrist registration is not enforced. However, these techniques are not without risks and involve considerable cost and parental time commitments for regular drug administration or lens care, as well as regular follow up consultations. Therefore, the decision to undertake an intervention should be made taking into due consideration all aspects of the treatment and the likely benefits to the child. Recently, some researchers have suggested that all myopic children should be immediately enrolled into an intervention [17]. Fang and co-workers even suggested that all children, regardless of their refractive status, should be treated with atropine [18]. However, in general, researchers and practitioners would recommend atropine only for myopes, rather than a prophylactic treatment in pre-myopes [19, 20].

It should be recognized that not all children presenting with low myopia will progress to high myopia, with only approximately 10% reaching -6.00 D or above [1]. As many will progress either slowly or not at all, undertaking a costly and possibly risky procedure may be neither necessary nor advisable. Therefore, the decision to intervene needs to be taken based on clinical evidence and following informed discussion with the parents. There have been very few reports of following up myopia progression in children before the introduction of an intervention. A recent study determined that observation of axial elongation (AE) over a 7-month period allowed identification of children experiencing rapid eye growth and recommended that such subjects should be immediately fitted with ortho-k lenses [21], whilst a period of observation was recommended for subjects displaying moderate or slow eye growth, taking age into account. However, the paper focused on changes in AE only, and did not consider relationship with changes in refractive error, which is a more frequently measured parameter, especially in smaller practices lacking more sophisticated equipment. Refractive error is also better recognized by non-practitioners and the general public as a marker for myopia. The current study aimed to further analyse the data from that study [21] by exploring the relation of AE and changes in refractive error, in terms of myopia (M) with ortho-k outcomes, with respect to justifying the actions suggested in the previous paper.

## Materials and methods

**Fig 1:**
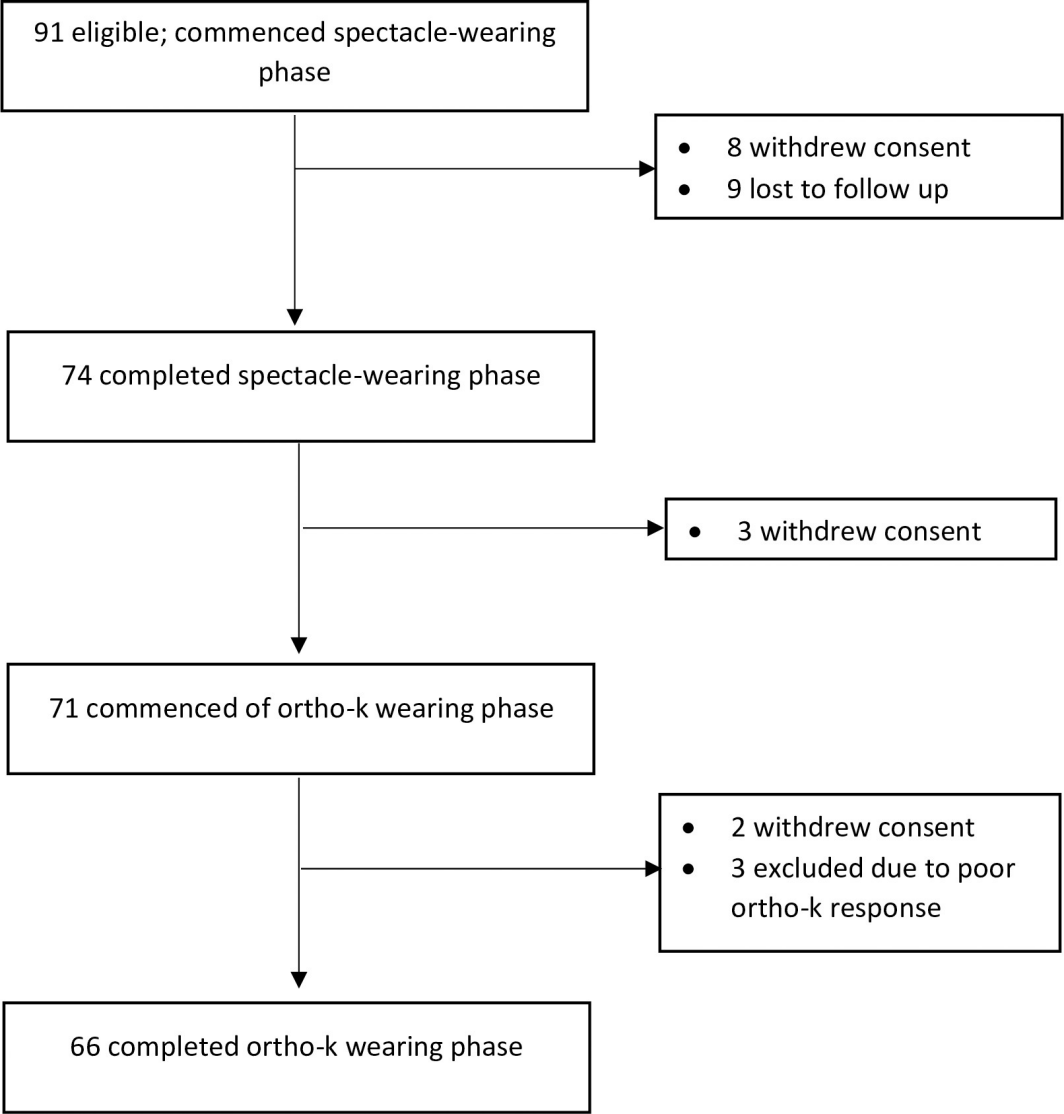
Study flowchart.

A study (Fig 1) was conducted at The Hong Kong Polytechnic University investigating the rate of AE before and after the use of ortho-k lenses (ClinicalTrials.gov,number NCT01236755) [21]. Briefly, subjective refractive errors and axial length (IOLMaster; Carl Zeiss Meditec, Inc., CA, US) after cycloplegia were determined for 66 myopic children aged from six to not more than 16 years, who had no previous experience in myopia control or contra-indication for ortho-k lens wear. The study was approved by the Ethics Committee of the School of Optometry of The Hong Kong Polytechnic University and followed the tenets of the Declaration of Helsinki. Consents was obtained from both parents and subjects prior to the commencement of the study. No control was required in this self-controlled case series study, as changes were reported in individual subjects. Bias was minimised by the standard protocol of study to control treatment given, examination procedures and examination schedule, and the primary outcomes (both axial length and the post cycloplegic subjective and objective refractive error) were performed by a masked examiner.

All subjects were recruited via advertisement at local newspapers. Measurements were performed before and after seven months of wearing single-vision spectacles (spherical lenses of refractive index 1.56; Founder Optical Company, Hong Kong) (Phase I), and after seven months of wearing ortho-k lenses (Menicon Z Night or Z Night Toric; NL Contactlenzen BV, Emmen, The Netherlands) made in super high oxygen permeable material (Dk 163; ISO/Fatt 189) (Phase II). All spectacles, ortho-k lenses and lens care products were provided for the subjects.

Subjects were grouped according to age: 14 Younger Children (YC) (6 to < 9 years), 36 Older Children (OC) (9 to < 13 years), and 16 Early Adolescent (EA) (13 to < 16 years) [21]: these numbers provided 99.8%, 99.9%, and 86.7% power to detect a significant change in AE between pre-treatment and treatment phases of the study in each age group (G*Power version 3.1.9.2).

Myopia progression was classified as slow (AE < 0.10 mm; M change < 0.29D), moderate (AE > 0.10 mm but < 0.20 mm; M change 0.29 to < 0.58D), or rapid (AE ≥ 0.20 mm; M change ≥ 0.58D), equivalent to < 0.50 D, 0.50 D to < 1.00 D, and ≥ 1.00 D increase in myopia over one year [22, 23], based on AE and M change in Phase I. The number of matches for each age group and progressor group was determined.

Outcomes of ortho-k treatment were evaluated with respect to age group and reduction in AE after ortho-k. Ortho-k treatment was determined as ineffectual (no change or an increase in AE was observed), not clinically significant (reduction in AE ≤ 0.05 mm), and significant benefit (reduction in AE > 0.05 mm). Based on the previous recommendations for management of myopes as initially presented [21], the outcome was compared with the expected benefit of ortho-k based on AE and M change.

### Data analysis

Data from the eye showing the greatest increase in axial length in Phase 1 was used for analyses. Where both eyes showed similar changes in axial length, the eye showing the greater increase in myopia was chosen. All subjects completed all study visits and no missing data was observed. Statistical analyses were performed using SPSS software version 23 (IBM corporation, NY, USA) with significance level of 0.05. Since distributions of M and M change were significantly different from normal (Shapiro-Wilk tests), parametric tests were used for testing AE, whereas non-parametric tests were used for testing change in M. For categorisation by AE, one-way ANOVAs was used to compare the baseline characteristics, whereas ANCOVAs were used to evaluate AE in each study phase and its change after ortho-k, adjusting for age, initial axial length, and initial M. Bonferroni corrections were used in the post-hoc analysis if significant main effects were found in ANCOVA. Kruskal-Wallis tests were used for categorisation by M change before and after ortho-k treatment. Mann-Whitney U tests with a significance level of 0.017 were used for post-hoc analysis if significant findings were observed in Kruskal-Wallis tests. The categorised rates (slow, moderate, or rapid) for AE and M change in the spectacle-wearing phase were compared using McNemar-Bowker test and Cohen’s kappa for all 66 subjects.

## Results

**Fig 2:**
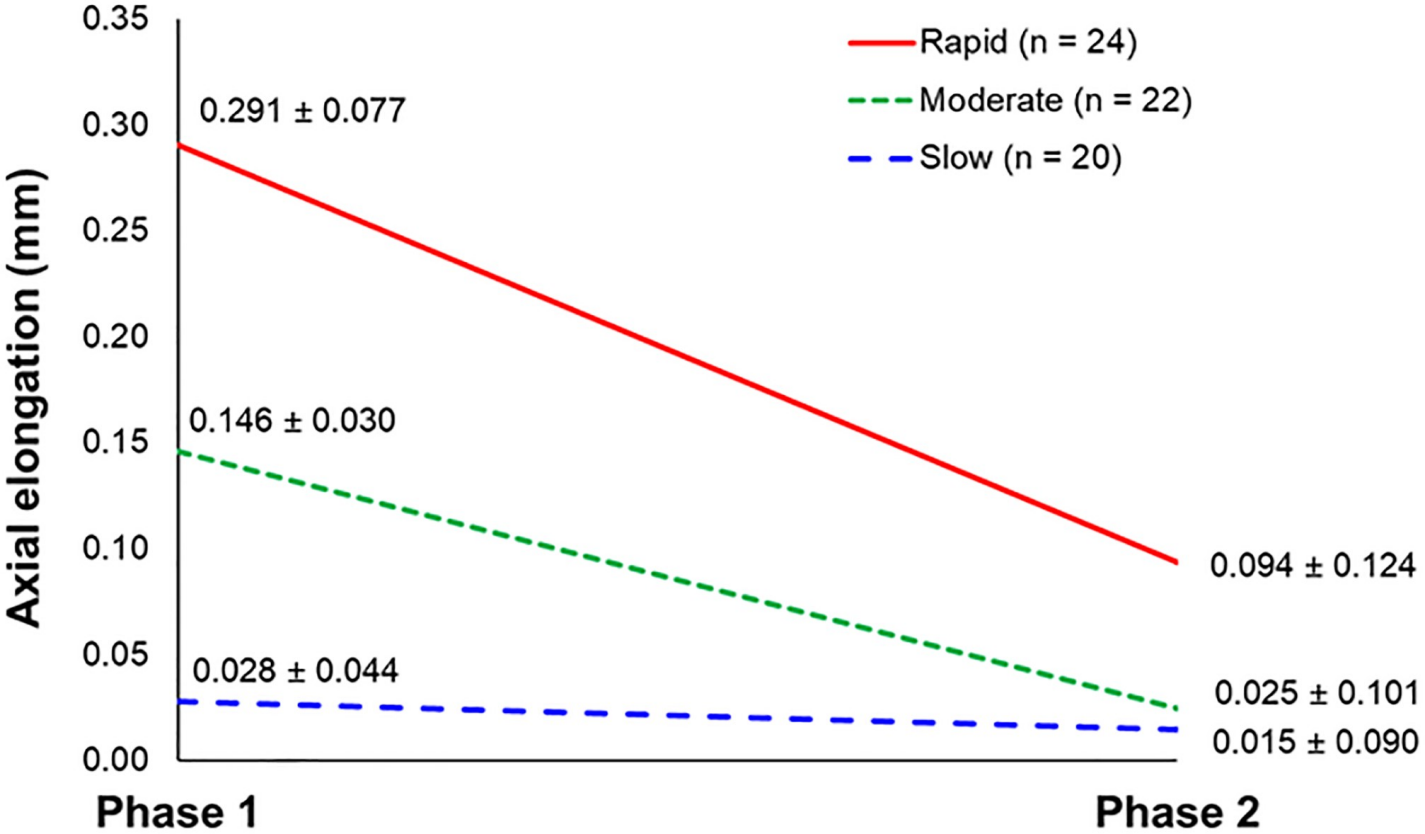
Mean (± standard deviation) axial elongation (AE) in Phases 1 (spectacle-wear phase) and 2 (orthokeratology phase) of various progressing groups, defined by AE in Phase 1.

At the commencement of ortho-k lens wear, there were no significant differences in myopia and axial length among the slow, moderate, and rapid progressors defined by AE and M change (p > 0.05), but subjects with rapid progression were younger than those with slow progression (p < 0.001) (Table 1). After ortho-k lens wear, rapid progressors had the greatest reduction in AE. In contrast, slow progressors had virtually no change in AE (Table 1 and Fig 2).

**Table 1:** Baseline data before orthokeratology lens wear, and axial elongation (AE) and change in myopia (M) before and after orthokeratology lens wear in subjects categorised by rate of myopia progression, defined by AE and change in M in the spectacle-wearing phase.

	End of spectacle-wearing phase					End of ortho-k-wearing phase	
	Age	Myopia (D)	Axial Length (mm)	Change in M (D)	AE (mm)	AE (mm)	AE Change (mm)
					(A)	(B)	(B-A)
**All subjects (n = 66)**	11.31 ± 2.26	-2.48 ± 0.82	24.53 ± 0.83	0.30 ± 0.34	0.163 ± 0.121	0.047 ± 0.112	-0.116 ± 0.122
**Category by AE**							
Rapid (R; n = 24)	9.48 ± 2.08	-2.31 ± 0.68	24.37 ± 0.97	-0.531 ± 0.406	0.291 ± 0.077	0.094 ± 0.124	-0.197 ± 0.116
Moderate (M; n = 22)	11.79 ± 1.48	-2.53 ± 0.91	24.46 ± 0.88	-0.284 ± 0.140	0.146 ± 0.030	0.025 ± 0.101	-0.121 ± 0.094
Slow (S; n = 20)	12.98 ±1.53	-2.61 ± 0.88	24.80 ± 0.48	-0.050 ± 0.208	0.028 ± 0.044	0.015 ± 0.090	-0.014 ± 0.074*
Between group analysis	***<0*.*001***^***#***^	0.453^***#***^	0.206^***#***^	***0*.*027^***	--	0.886***^***	***<0*.*001^***
*Post-hoc*	S vs. M = 0.090	NA	NA	*S vs M = 0*.*674*	--	NA	***S vs M = 0*.*002***
	***S vs R < 0*.*001***			***S vs R = 0*.*024***			***S vs R < 0*.*001***
	***M vs R < 0*.*001***			*M vs R = 0*.*435*			***M vs R = 0*.*010***
**Category by change in M**							
Rapid (F; n = 5)	7.95 (7.69–8.13)	-2.25 (-3.25 –-0.75)	24.17 (23.18–25.11)	-1.25 (-1.75 –-0.75)	0.37 (0.30–0.47)	0.19 (-0.04–0.31)	-0.27 (-0.34 –-0.06)
Moderate (M; n = 15)	10.41 (6.49–15.14)	-2.50 (-4.00 –-1.25)	24.57 (23.32–26.21)	-0.50 (-0.50 –-0.50)	0.22 (0.08–0.41)	0.06 (-0.11–0.35)	-0.15 (-0.49–0.00)
Slow (S; n = 46)	11.96 (8.15–15.77)	-2.25 (-4.25 –-0.75)	24.64 (22.74–26.44)	-0.25 (-0.25 –+0.25)	0.12 (-0.07–0.42)	0.04 (-0.17–0.27)	-0.07 (-0.33–0.11)*
Kruskal-Wallis Tests	***<0*.*001***	0.464	0.579	--	***<0*.*001***	***<0*.*001***	***0*.*008***
*Post-hoc*	***S vs*. *M = 0*.*020***	NA	NA	--	***S vs M < 0*.*001***	***S vs M < 0*.*001***	S vs M = 0.025
	***S vs R < 0*.*001***				***S vs R < 0*.*001***	***S vs R < 0*.*001***	***S vs R = 0*.*012***
	M vs R = 0.066				***M vs F = 0*.*004***	***M vs R < 0*.*001***	M vs R = 0.395

* Clinically insignificant (<0.09 mm (equivalent of <0.25D))

#: One-way ANOVA

^ ANCOVA adjusted for initial age, myopia and axial length.

**Fig 3:**
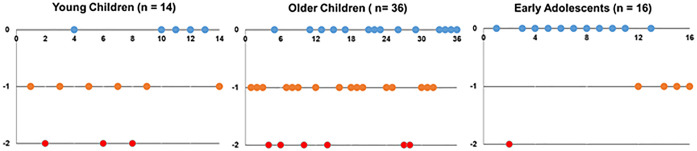
Matching of categories by axial elongation and changes in myopia in the three different age groups. Matching pairs are indicated by ‘0’. Progression rate was under-estimated either by 1 category (indicated by ‘-1’) (i.e. ‘Rapid’ to ‘Moderate’ or ‘Moderate’ to ‘Slow’), or 2 categories (indicated by ‘-2’) (i.e. ‘Rapid’ to ‘Slow’).

Categorisation of the rate of progression using AE and M change was in poor agreement (Cohen’s Kappa = 0.008; Bowker test, p < 0.001), such that about 77% and 42% of subjects classified as moderate and rapid progressors by AE, respectively, were misclassified as ‘slow’ progressors by M change (Table 2). Matched categorisation reduced from 95% for slow progressors, to 23% and 21% for moderate and rapid progressors, respectively (Table 2). For the three individual age groups, matching pairs increased from 36% and 39% in the YC and OC groups, respectively, to 69% in the EA group (Fig 3).

**Table 2:** Agreement in categorisation by axial elongation (AE) and change in myopia (M) in spectacle-wearing phase (Bold indicates matched categorisation).

**All subjects**	**AE Slow (n = 20)**	**AE Moderate (n = 22)**	**AE Rapid (n = 24)**
M Slow (n = 46)	**19 (95.0%)**	17 (77.3%)	10 (41.7%)
M Moderate (n = 15)	1 (5.0%)	**5 (22.7%)**	9 (37.5%)
M Rapid (n = 5)	0	0	**5 (20.8%)**
**(A) Young children**	**AE Slow (n = 0)**	**AE Moderate (n = 1)**	**AE Rapid (n = 13)**
M Slow (n = 4)	**0**	1 (100%)	3 (23.1%)
M Moderate (n = 5)	0	0	5 (38.5%)
M Rapid (n = 5)	0	0	**5 (38.5%)**
**(B) Older children**	**AE Slow (n = 10)**	**AE Moderate (n = 17)**	**AE Rapid (n = 9)**
M Slow (n = 28)	**9 (90.0%)**	13 (76.5%)	6 (66.7%)
M Moderate (n = 8)	1 (10.0%)	**4 (23.5%)**	3 (33.3%)
M Rapid (n = 0)	0	0	**0**
**(C) Early adolescents**	**AE Slow (n = 10)**	**AE Moderate (n = 4)**	**AE Rapid (n = 2)**
M Slow (n = 14)	**10 (100%)**	3 (75%)	1 (50%)
M Moderate (n = 2)	0	**1 (25%)**	1 (50%)
M Rapid (n = 0)	0	0	**0**

Outcomes of ortho-k treatment are shown in Table 3. Myopia control was considered to be of significant benefit if reduction in AE was more than 0.05mm in 7 months (or equivalent to reduction in myopia of 0.25D per year). Among the 24 rapid progressors categorised by AE, 21 (87.5%) showed significant benefit after 7 months of ortho-k lens wear (Table 3), including the five subjects correctly identified by M change. About 73% of moderate progressors defined by both variables, would benefit from ortho-k. Percentage of slow progressors with significant beneficial effect changed from 0% when categorised by AE to 46% when categorised by M change. This result may be related to the under-estimation of progression based on M change. Beneficial effect was observed among the 27 (59%) subjects with moderate and rapid AE, but mis-classified into slow by M change.

**Table 3:** Details of outcomes on myopia control of orthokeratology lens wear of children in different age groups classified by axial elongation (AE) and changes in myopia (M). Outcomes were classified into ‘Ineffectual’, ‘Not clinically significant’ and ‘Significant benefit’ according to the change in AE after switching from spectacle-wear to orthokeratology lens wear.

Age groups	Young Children (6 –< 9 y)	Older Children (9 –< 13 y)	Early Adolescents (13 –< 16 y)	Young Children (6 –< 9 y)	Older Children (9 –< 13 y)	Early Adolescents (13 –< 16 y)	Young Children (6 –< 9 y)	Older Children (9 –< 13 y)	Early Adolescents (13 –< 16 y)
	**Classification by AE**								
	**Slow (n = 20)**			**Moderate (n = 22)**			**Rapid (n = 24)**		
Ineffectual	0	5	7	0	2	0	1	0	0
Not clinically significant	0	5	3	0	4	0	2	0	0
Significant benefitted	0	0	0	1	11	4	10	9	2
	**Classification by change in M**								
	**Slow (n = 46)**			**Moderate (n = 15)**			**Rapid (n = 5)**		
Ineffectual	0	6	7	1	1	0	0	0	0
Not clinically significant	1	8	3	1	1	0	0	0	0
Significant benefitted	3	14	4	3	6	2	5	0	0

Ineffectual: increase in AE ≥ 0.00 mm.

Not clinically significant: reduction in AE > 0.00 to ≤ 0.05 mm (equivalent to ≤ 0.25D per year).

Significantly benefitted: reduction in AE > 0.05mm (equivalent to > 0.25D per year).

## Discussion

The current study used the data from the subjects’ eye demonstrating greater AE during spectacle-wearing phase. Eyes showing rapid AE did not necessarily display corresponding M change. In this study, 46 out of 66 subjects (70%) showed a greater increase in axial length and myopia in the same eye in Phase I. Reanalysis of the data using the faster eye for each parameter resulted in similar findings and so, this report has concentrated on the use of the data from the eyes demonstrating greater AE as it allowed the investigation of the correlation between AE and M change.

This study investigated whether the success of an intervention of ortho-k for myopia control can be predicted based on a pre-treatment observation period. This approach is unusual, as in most prospective studies, myopes are recruited and assigned into groups receiving, with or without randomization, either a myopia control intervention or a control treatment without a run-in period [7–9, 24, 25]. Most of the subjects in the cross-over study by Swarbrick and co-workers [26] were early adolescents with slow progression. Pauné and co-workers [27] also recruited older myopic children aged 9–16 years, but slow progressors (increase in myopia < -0.30D/year) were excluded. The differentiation of subjects into rapid, moderate, or slow progressors before commencement of ortho-k lens wear and determining the effectiveness of slowing AE and, therefore, the benefit of the treatment is, to our knowledge, a novel approach. The current study found that the benefits of ortho-k intervention was greatest for those having rapid progression in AE. For slow progressors, the effect of the intervention was not clinically significant, thus rendering such an intervention inappropriate (see Fig 2). This emphasizes the importance of determining the rate of axial growth before commencing an intervention.

It is recognized that in many small practices, ocular biometry is not performed routinely due to lack of equipment, leading to reliance on refractive error measurement. This can be misleading, as M change was poorly correlated with AE and, thus, may not reflect the actual risk in high myopia, which is increased axial length. In this study, M change and AE were fairly well correlated for slow progression. Thus, subjects with slow myopia progression (by AE and M change) would not be considered as candidates for immediate therapy, as suggested in the previously published guidelines [21], although continued monitoring is, of course, important as it is recognized that myopia progression rates may vary over a period of years [28]. The combination of the initial and subsequent monitoring period would usually allow for slow M change to catch up with moderate or rapid changes in AE. A few subjects with slow AE showed moderate changes and one, a rapid change, in refractive error, but such changes may not be axial in origin [29]. An immediate decision simply based on change in refractive error, but not the status of axial progression, would lead to these children entering into a myopia control intervention, with little expectation of improvement, while increasing their risk of adverse effects [11], as well as time and economic burdens [30, 31].

Matching in categorisation was reduced for subjects with moderate and rapid AE (Table 2), making M change a poor predictor for necessity of intervention. For subjects with rapid AE, 41.7% were misclassified as slow by M change. Appropriate intervention would have been delayed in such subjects if diagnosis was based on M change alone (Table 2). The discrepancies in categorisation by AE and M change can clearly be observed in Fig 3. Overall, categorisation by M change tended to be lower than that by AE and this was particularly worrying for YC subjects categorised as rapidly progressing by AE, but slow by M change.

This study has shown that further monitoring of slow progressors determined by AE can reduce imposing unnecessary treatment, as none of the slow progressors benefited from the myopia control intervention. Although, axial length measurement is recommended in myopia control research [31], it is still not a routine procedure in clinical practice [17]. As seen in this study, M change may not reflect that of AE. However, excessive AE, even if not associated with change in refractive error, still increases the risk of later pathological changes (e.g. retinal tear or detachment) [32], due to thinning of the parafoveal region, it, therefore, should be prevented. The current findings show that ocular biometry is not only useful in determining the risk of myopia pathology, but also an important instrument to truly identify the children at risk from those who are not. It is encouraging that manufacturers are moving to production of practice-friendly all-in-one equipment to measure both axial length and refractive error. In addition, auto-refractors with improved accuracy and reliability, especially for children due to their poor cooperation and high range of accommodation, could improve the precision of refraction [33]. However, this alone will not suffice as accurate records of progression are needed to make an appropriate decision. For small practices not properly equipped for modern myopia management, practitioners may consider referral and co-management with other practitioners for timely myopia management. For instance, children may receive frequent follow up on the change in refractive error and update their prescription (spectacles or contact lenses) in their local practitioners, coupled with six or 12 monthly cycloplegic examination of AE in the collaborating practice. However, in the long term, it may be appropriate for practitioners to consider updating their equipment to meet advancement in myopia management.

In myopia control management, it is also prudent for practitioners to maintain close contact with patients in order to develop a good and timely individual management plan, as sometimes the decision for myopia control intervention may be delayed, with the practitioner adopting a ‘wait and see’ approach. A ‘wait and see’ approach, otherwise known as “watchful waiting” or “active surveillance” for slow progression of disease is not unknown in other branches of medicine. This strategy has been used for procedures in which the risk of adverse effects may outweigh benefits to slowly or non-progressing patients, including for those with depression, otitis media, inguinal hernia, prostate cancer, and non-symptomatic kidney stones [34].

Ideally, evaluation of refractive status and eyeball length in children should commence as early as possible and not be delayed until onset of myopia. This is of the utmost importance among the children aged 6 to 9 years, as they are at the highest risk from rapid progression. This provides the necessary history showing the progression of refractive and ocular changes and obviates the necessity for the 6-month pre-intervention monitoring suggested. Development of myopia even in at-risk children may take several years, as shown by Mutti and co-workers [5], who monitored refraction and axial length over a 7-year period. Currently, most children are only examined when starting school or when a problem is already apparent. Parents should be encouraged to commence regular eye examination for their children at an early age.

**Fig 4:**
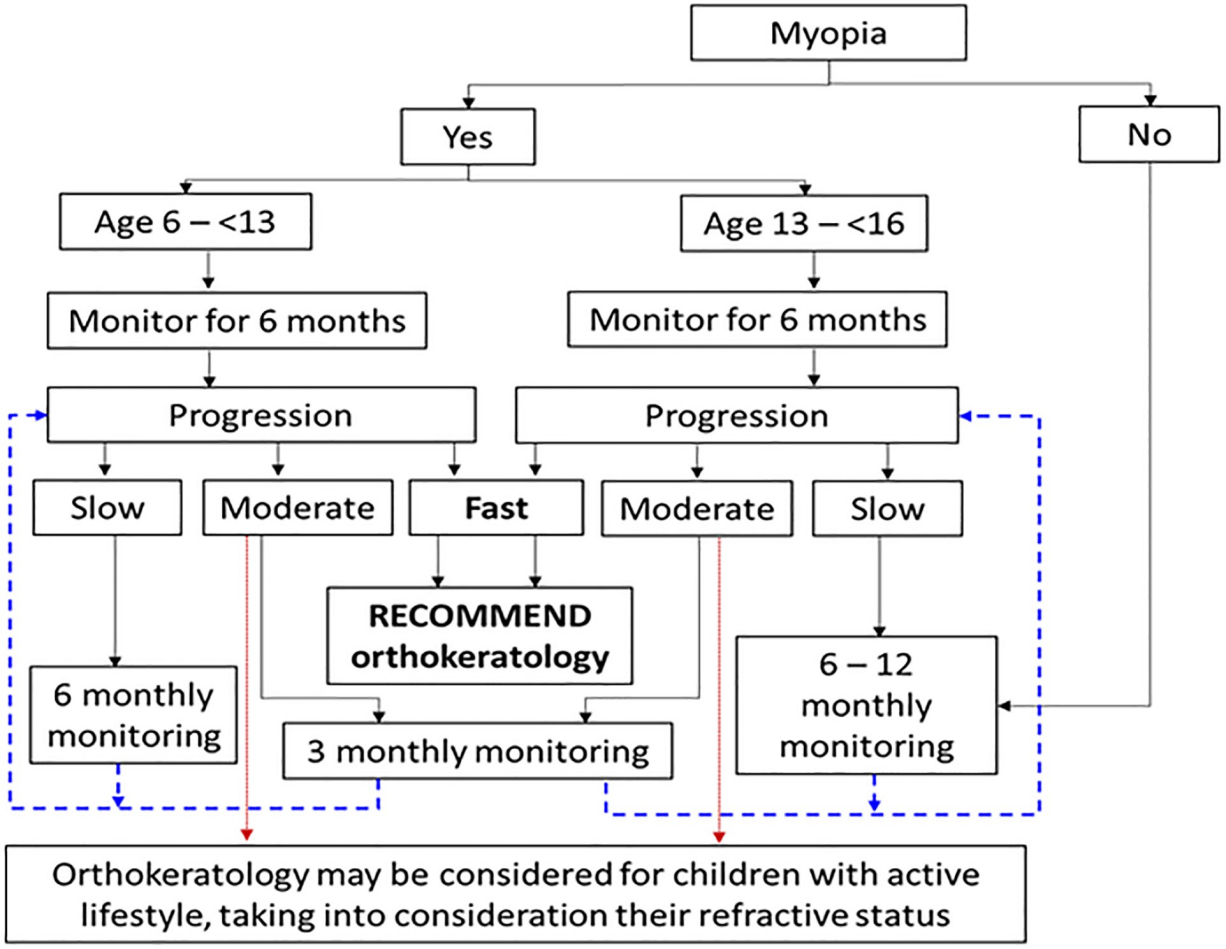
Revised decision tree for myopia control management for children aged 6 to < 16 years.

As shown in the results, subjects with rapid progression by AE and M change, would virtually all benefit from ortho-k. Thus, the use of the guidelines to immediately recommend therapy for rapid progressors would be appropriate and beneficial. Moderate AE progression was most frequently observed in the OC group (17/22; 77.3%). Of these OC subjects, 11 (65%) received significant benefit from ortho-k and the remaining six (35%) did not benefit from the treatment. One YC and four EA subjects demonstrated moderate AE all benefitting from the intervention. Similarly, EA subjects with moderate M change also benefitted from ortho-k. Based on the previous guidelines for management of myopic children [25], in the absence of other indication (e.g. active lifestyle) for myopia control using ortho-k, these moderate progressors would receive 3-monthly (for YC and OC groups) or 6-monthly (for EA group) additional monitoring while using single-vision spectacles. Thus, modification to our initial recommendation was indicated by the current findings, such that early treatment or more frequent monitoring period would be more appropriate for the moderate progressors, especially for EA subjects, such that children aged below 16 with moderate progression should be monitored at intervals (see Fig 4), rather than the six initially suggested in our guidelines [25]. A 3-month waiting period will not significantly change the risk for development of high myopia, but would determine whether an intervention is appropriate. As EA subjects with moderate and rapid AE may be misclassified into slow by M change, the monitoring period for EA with slow progression was also revised from 12-monthly to 6- to 12-monthly (Fig 4).

The use of guidelines to facilitate myopia control intervention decision-making is prudent, because most interventions carry a modicum of risk and time commitment, and all impose a substantial financial outlay. Although some recent recommendation for initiation of myopia control at the onset of myopia [20, 23], the current study shows that the decision should be taken dependent on the actual need and likelihood to benefit from the intervention. This is particularly the case for EA subjects for whom benefits are far less likely.

As with many studies requiring long-term commitment to monitoring before therapy, the current study suffers from a relatively small sample size, especially for YC and EA groups. However, a post-study power calculation indicated that the sample size of each group did provide adequate power to detect a significant difference. Parents of young children either had concerns about lens wear at an early age or were eager to immediately embark on therapy. In contrast, early adolescents may already have a history of myopia control intervention. Whilst the guidelines were developed based on the results of this study, which utilized ortho-k as an intervention, they are likely to be relevant for other types of interventions in order to maximize benefits and to reduce unnecessary risks and financial burdens. Similar studies would be necessary to confirm these recommendations for other interventions.

## Conclusion

Prediction of myopia progression by change in M has poor correlation with the need for intervention, emphasizing the importance of axial length measurements in the management of myopia. In-depth analysis of benefits of ortho-k to myopic children with varying rates of AE demonstrated that immediate intervention for those with rapid progression generally resulted in significant benefit, whilst a ‘wait and see’ approach appears to be a reasonable practice for moderate and slow progressors.

## Supporting information

S1 File (DOC)

S2 File TREND statement checklist. (PDF)
