# Supplementary material for: Categorisation of myopia progression by change in refractive error and axial elongation and their impact on benefit of myopia control using orthokeratology
Source: PLoS One. 2020 Dec 29;15(12):e0243416. doi: 10.1371/journal.pone.0243416 (PMC7771698; doi:10.1371/journal.pone.0243416)
Supplement: S1 File — (DOC) [file pone.0243416.s001.doc]

**Discontinuation of Orthokeratology on Eyeball Elongation (DOEE) Study**

**Proposal by**

**Dr Pauline Cho**

**School of Optometry**

**The Hong Kong Polytechnic University**

***Introduction***

Children wearing orthokeratology (ortho-k) have been shown to have slower rate of myopic progression than those wearing single-vision spectacles (Cho et al. 2005) or soft lenses (Walline et al. 2009). Both studies showed that the effect of myopic control was most significant during the first six months of the treatment. It is unknown whether the myopic control effect would dissipate upon the discontinuation of the treatment or whether the myopic control effect only happened in the first six month of lens wear and was maintained thereafter.

Parents of children wearing ortho-k always have questions such as ‘What is the current amount of myopia of the children’, ‘How long do my child need to wear the lenses to slow progression of myopia’ or ‘My child has been on the treatment for a few years. When can s/he stop the treatment?’. In order to answer these questions, to understand the effect of ortho-k more thoroughly, there is a need for a study evaluating the effect of discontinuation of ortho-k treatment on myopic control. This knowledge is necessary and important as patients/parents have concerns about the permanent dependency on ortho-k once they enrolled in the treatment. Would only 6 months ortho-k lens wear (and then switch to spectacles or contact lenses) be adequate for myopic control or does the child need to continue lens wear until s/he is of certain age?

Younger myopic children (aged 6-10 years old) may have the faster increase in myopia than old myopic children (aged 11-15 years old), i.e. the rate of myopic progression may be different in different age groups and in children with different refractive status (Edwards 1999; Fan et al. 2004; Cheng et al. 2007). Although the refractive correction with ortho-k has been well documented, it is unknown whether the efficiency and reversibility of ortho-k for myopic reduction as well as myopic control are similar in children of different age and refractive groups.

***Objectives***

* The primary objectives of this study will be to compare

- eyeball elongation in existing ortho-k subjects who stopped lens wear with subjects who continued ortho-k lens wear and control subjects wearing glasses (Experiment 1)
- rates of change in axial length in younger and older myopic children when corrected with ortho-k and when ortho-k was discontinued (Experiment 2)
- rates of change in axial length in children with faster and slower myopic progression rates (Experiment 2)
- change in corneal parameters during and after discontinuation of lens wear (Experiment 3)

***Study design***

Single masked, prospective study consisting of three experiments.

**Experiment 1**

| **Control** | | | | | | | | | | | | | | | | | | | | | | | | | | | | |
| --- | --- | --- | --- | --- | --- | --- | --- | --- | --- | --- | --- | --- | --- | --- | --- | --- | --- | --- | --- | --- | --- | --- | --- | --- | --- | --- | --- | --- |
| **Continue spectacle-wear** | | | | | | | | | | | | | | | | | | | | | | | | | | | | |
| **Group OKc** | | | | | | | | | | | | | | | | | | | | | | | | | | | | |
| **Continue orthokeratology** | | | | | | | | | | | | | | | | | | | | | | | | | | | | |
| **Group OKd** | | | | | | | | | | | | | | | | | | | | | | | | | | | | |
| **RS** | **SPECTACLES** | | | | | | | | | | | | | **RS** | **ORTHOKERATOLOGY** | | | | | | | | | | | | | |
|  | | | | | | | | | | | | | | | | | | | | | | | | | | | | |
| 1 |  | 2 |  | 3 |  | 4 |  | 5 |  | 6 |  | 7 |  |  |  | 9 | |  | 10 |  | 11 |  | 12 |  | 13 |  | 14 |  |
|  | | | | | | | | | | | | | | | | | | | | | | | | | | | | |
| I-0 | I-1 |  |  |  | I-3 |  |  |  |  |  | I-6 |  | I-7 |  | II-1 | |  |  |  | II-3 |  |  |  |  |  | II-6 |  | II-7 |

**Experiment 2**

|  | **SPECTACLES** | | | | | | | | | | | | | **RS** | **ORTHOKERATOLOGY** | | | | | | | | | | | | |
| --- | --- | --- | --- | --- | --- | --- | --- | --- | --- | --- | --- | --- | --- | --- | --- | --- | --- | --- | --- | --- | --- | --- | --- | --- | --- | --- | --- |
| Month (estimates) |  | | | | | | | | | | | | | | | | | | | | | | | | | | |
| 1 |  | 2 |  | 3 |  | 4 |  | 5 |  | 6 |  | 7 |  | 8 |  | 9 |  | 10 |  | 11 |  | 12 |  | 13 |  | 14 |  |
| Visit |  | | | | | | | | | | | | | | | | | | | | | | | | | | |
| I-0 (baseline) | I-1 |  |  |  | I-3 |  |  |  |  |  | I-6 |  | I-7 |  | II-1 |  |  |  | II-3 |  |  |  |  |  | II-6 |  | II-7 |

Cycloplegic data collection visits Non-cycloplegic data collection visits

I-0 I-3, II-3

I-1, II-1 I-6, II-6

I-7, II-7

**Figure 1. Data collection visits in the experimental phases in the discontinuation study.**

*Data collection visits*

*Phase I*

- Visit I-0 – Commencement of Phase I for all subjects; Commencement of RS period for Group OKd (Experiment 1)
- Visits I-1, I-3, I-6, I-7: about one, three, six and seven months after Visit I-0

*Phase II*

- Visit I-7 – Commencement of Phase II for all subjects; Commencement of RS period for Group OKd (Experiment 1) and all subjects in Experiment 2
- Visits II-1, II-3, II-6, II-7: about one, three, six and seven months after Visit I-7

**Experiment 1.** *Discontinuation of lens wear in existing ortho-k children*

The objective of this study is to compare eyeball elongation in existing ortho-k subjects who stopped lens wear with subjects who continued ortho-k lens wear and control subjects wearing glasses.

Figure 1 shows the required visits for the duration of the experiment for each group of subjects.

In this experiment, subjects who have completed the ROMIO, TO-SEE and HM-PRO studies will be invited to participate. All spectacle-wearing control subjects will be invited to continue as control subjects. Ortho-k subjects will be randomly assigned into Groups OKc or OKd.

All subjects will be followed up for a period of 14 months.

- Group C will continue to wear spectacles for the duration of the experiment
- Group OKc will continue lens wear for the duration of the experiment
- Group OKd will be required to discontinue lens wear for 6 (+1*) months (and wear single-vision spectacles Phase I) and then resume ortho-k lens wear for another 6 (+1*) months (Phase II). *The first month (*) of each phase (See Figure 1), where indicated, is the RS (Refraction Stabilization) period to allow stabilization of refractive errors.*

*Group OKd*

At the beginning of Phase I (Spectacle-wear phase), subjects will be required to wear single-vision spectacles to aid distance vision in the RS period. They are required to use their old spectacles which should be +/– 0.50DS in sphere and +/– 0.50 DC in cylinder from the refraction determined at the time of visit, otherwise, new spectacle lenses will be ordered. They will be followed up weekly until stabilization of refractive errors has been achieved and the stabilized refraction will be used for the prescription of the spectacles which will be delivered at Visit I-1. Once the refraction has stabilized, all children will be required to wear fully-corrected spectacles in the daytime during Phase I. They will be excluded if they regularly use any kind of contact lenses during Phase I.

New ortho-k lenses for these subjects will be ordered one month before the end of Phase I (ie. at Visit I-6) and dispensed at the end of Phase I (Visit I-7) before commencing Phase II (see Table 1).

In Phase II (Ortho-k phase), subjects are required to wear the ortho-k lenses every night unless otherwise instructed by their examiner. They need to attend the ortho-k aftercare visits as scheduled. Complimentary contact lens solutions and lens accessories (Table 2) will be supplied during the treatment. They have to keep an ortho-k journal everyday. Single-vision spectacles may be needed to aid distance vision during the RS period.

Cycloplegic data collection will be conducted at the beginning of each phase, at the end of RS period (for Group OKd only) and at the end of each phase. Subjects will be required to come back for the regular non-cycloplegic data collection visits after RS period. Change in axial length in the 6 months following stabilization of refractive errors for each Phase will be determined and compared within subjects and between groups. The whole study period for each subject will last for about 14 months.

*Group C and Group OKc*

Subjects will wear their habitual spectacles/ortho-k lenses at the commencement of Phase I, before Visit I-1. New glasses will be made based on Rx determined at Visit I-0 and delivery of new glasses will be at Visit I-1 and if indicated (based on data collected at Visit I-6), at Visit I-7 before commencing Phase II. Note that there is no RS period for these two groups of subjects, but cycloplegic and non-cycloplegic data correction visits will be same as Group OKd.

**Experiment 2.** *Discontinuation of lens wear in new ortho-k children*

The objective of this experiment is to compare axial elongation in younger and older children during ortho-k treatment and after discontinuation of ortho-k lens wear. Forty-five children (aged 6-10) and 45 older children (aged 11-15) who are myopic of 1.50 to 4.50D and who intend to have overnight ortho-k for myopic control will be recruited via advertisement in newspaper or by referral. They should have no previous experience in rigid lens wear and no prior experience in myopic control treatment. The inclusion criteria are listed in Table 3.

Similar to Experiment 1, the subjects will have to participate in a two-phase experiment (see Figure 1).

At the commencement of Phase I (Visit I-0), the subjects’ baseline data will be collected and prescriptions for new spectacles will be determined. New spectacles will be dispensed at Visit I-1. They will be required to use the spectacles in the daytime for the following 6 months (the rest of Phase I). Ortho-k lenses (Menicon Z Night or Menicon Z Night Toric) will be ordered 1 month before the end of Phase I (i.e. Visit I-6), after they have learnt lens handling. Ortho-k lenses, contact lens solutions and daily log will be dispensed at the end of Phase I (Visit I-7) before commencing Phase II (see Table 1). They are required to wear the ortho-k lenses every night in Phase II unless otherwise instructed by their examiner.

Visits schedule and methodology will be the same Experiment 1 (Figure 1 & Table 1).

The study period for each subject will be about 14 months (including 1 month for baseline data collection).

**Experiment 3.** Refractive and corneal changes, including corneal thickness, corneal hysteresis, and corneal resistant factor during ortho-k treatment and the discontinuation of the treatment will be determined. These will be compared in new and existing ortho-k subjects and children of different ages. Information will be collected from subjects participating Experiments 1 and 2.

***Subjects***

Children between 6-15 years old will be recruited. Informed consent will be obtained from both the parents and the children at the beginning of the study.

- For Experiment 1, 80 ortho-k and 40 control subjects who have completed the 2-year myopic control studies (ROMIO, TO-SEE & HM-PRO) will be recruited. They were 6-12 years old when they joined the myopic control study and will be 8-14 years old when they commence this part of the study. Ortho-k subjects will be randomized into Group OKc where subjects will continue lens wear for another 12 months and Group OKd where subjects will be required to cease lens wear for 6 (+1*) months and wear single-vision spectacles and then resume ortho-k lens wear for another 6 (+1*) months.
- In Experiment 2, two groups of 45 children of aged 6-11 and 12-15 years old, myopia of at least 1.50D and astigmatism of at most 2.50D, and who intend to have overnight ortho-k for myopic control and have no contra-indication for ortho-k lens wear will be recruited via advertisement in newspaper or by referral. Subjects will be monitored for 6 months before they are fitted with ortho-k lenses which they have to wear for 6 (+1*) months.

***Examiners***

One examiner (E1) will be responsible for lens fitting and monitoring the subjects and will therefore be involved in every visit. The masked examiner (E2) will be responsible for collection of the primary outcome measures, will be masked to the treatment received by each subject, and will only be involved in the data collection visits. Data entries and analysis will be made by another independent examiner (E3). E1 will also be masked to the AL measurements of each subject.

***Examination procedures***

Table 4a and 4b list the test procedures and instruments used respectively in this study.

*Experiment 1*

The examiners will perform all test procedures including

- subjective
- objective refraction
- high/low contrast logMAR visual acuity assessment
- corneal topography
- slit-lamp biomicroscopy
- axial length measurement** (Cycloplegic and non-cycloplegic examination)
- corneal pachymetry
- non-contact intraocular pressure measurement
- corneal biomechanics assessment
- peripheral refraction (10° and 20°) (Cycloplegic examination)
- aberration (Cycloplegic examination)

Cycloplegia will be used on each subject at the cycloplegic data collection visits (0.5% alcaine followed by 1% tropicamide and 1% cyclopentolate).

**Only the masked examiner performs axial length measurements. Masked examiner will NOT perform any other procedures.

*Experiment 2*

Same as Experiment 1.

*Data collection visits*

Primary outcome measures will be determined in these visits, i.e. axial length. There are two types of data collection visits – cycloplegic data collection visits and non-cycloplegic data collection visits. The cycloplegic data collection visits are performed at the baseline, at end of the RS period, and 7 months after the beginning of each phase (see Figure 1 and Table 1). The cycloplegic data collection visit at the end of RS period will be conducted one week after stabilization of refraction has been achieved for OKd subjects in both Phases I and II in Experiment 1 and all ortho-k subjects in Phase II of Experiment 2.

The non-cycloplegic data collection visits are conducted every 2-3 months after RS period. In order to minimize the effect of diurnal variation, the data collection visits will be scheduled at about the same time of the day for each visit.

*Ortho-k aftercare visits*

For ortho-k subjects only. Regular ortho-k aftercare visits will be arranged upon delivery of ortho-k lens wear to ensure healthy and safe ortho-k lens wear. The ortho-k effect will be reviewed one night, one week, two weeks, three weeks, one month, and every 2-3 months after commencing lens wear. The weekly visits in the first month will only apply during the RS period.

- Commencement of lens wear. The night when the ortho-k treatment commenced/resumed. It shall be within 14 days after the last data collection visit.
- The first overnight visit. The following morning after commencing lens wear. It is within 2 hours after waking and children should wear the lenses to our clinic.
- One week aftercare visit. Seven days (± 3 days) after commencing lens wear, 2 hours after waking.
- Two weeks aftercare visit. Fourteen days (± 3 days) after commencing lens wear, 2 hours after waking.
- Three weeks aftercare visit. Twenty-one days (± 3 days) after commencing lens wear, 2 hours after waking.
- One month aftercare visit. Twenty-eight days (± 3 days) after commencing lens wear. Can be in the morning or in the afternoon.
- The three monthly after care visits. It will coincide with the data collection visits.

*Visits during RS period (Group OKd in Experiment 1 and all subjects in Phase II in Experiment 2)*

Weekly (± 3 days) review of refraction, corneal shape and ocular conditions once the ortho-k treatment has stopped. It usually consists of 4-6 visits, depending on the initial refractive errors of the subjects. Refraction is considered stabilized when the change in refractive sphere and refractive cylinder in manifest refraction in two consecutive visits is 0.25 D or less and cycloplegic examination (cycloplegic data collection visit at end of RS period) will be arranged in the following week.

***Ortho-k lenses, solutions and spectacles lenses***

*Ortho-k lenses*

Menicon Z Night and Night Toric lenses will be used in this study. Lenses to be ordered will be determined by the Easy Fit software (based on topographic data captured by the Medmont corneal topographer, the manifest refractive error and the corneal diameter). Full correction will be attempted for myopia ≤ 4.50D and partial correction targeted for 4.00D will be applied for myopia ≥ 4.50D.

- All ortho-k subjects who agreed to be randomized in Experiment 1 and all subjects in Experiment 2 will be provided with the required ortho-k lenses during the study period(s) as indicated.
- Subjects who refused to be randomized into Group OKd can participate in DOEE study as subjects in Group OKc if they pay for the lenses used during the experimental period (these will not be complimentary lenses but will be ordered via our clinic, ie. payment of lenses will go to our Optometry Clinic). They have to agree to return for the scheduled aftercare and data collection visits and each subject will receive a pair of complimentary lenses at the end of the study period.

*Contact lens solutions & accessories*

All the solutions used in this study are shown in Table 2. All solutions have to be replaced at least once every 2 months.

- Group OKc – Subjects will be required to purchase all solutions required (except for O2 Care) from our Clinic
- Group OKd in Experiment 1 and all ortho-k subjects in Experiment 2 –Complimentary contact lens solutions will be provided
- All subjects will be given complimentary flat lens cases, SP vial for weekly protein removal and O2 Care daily cleaning during the study period.

*Spectacle lenses*

Spectacles are required during the discontinuation period and may be needed in the first 2 weeks of the lens wear period. Subjects will be asked to keep their habitual spectacles. Complimentary spectacles lenses will be provided if indicated, provided that subjects use their own frames. New spectacle lenses will be prescribed once the refraction has stabilized during the RS period (where appropriate). Low refractive index *spherical* lenses will be prescribed for low myopes (myopia < 4.50D) and high refractive index lenses will be used for high myopes (≥ 4.50D) and high astigmatism (≥ 2.00DC).

***Termination of the study***

Subjects will be requested to withdraw from the study if any of the following occurs:

1. Persistent corneal staining (> Grade 2 in Efron’s scale)
2. Unaided monocular vision worse than 6/9 in either eye (only for full correction ortho-k subjects only)
3. Non-compliance with study protocol (e.g. wear contact lenses during the discontinuation period or rarely use ortho-k lenses during the lens wear period)

***Consistency of management***

- Standard fitting protocol will be used to minimize the effect of inter-practitioner difference.
- Both oral and written instructions will be given to the subjects to increase the consistency in lens handling as well as patient compliance.
- Subjects will be asked to keep an ortho-k journal to keep track of lens wear schedule, incidence of lens binding and problems encountered by the subjects. This information will be useful for the assessment of compliance and helpful to the practitioners in giving proper advice in the presence of complication.
- Contact lens solutions will be given and/or purchased (depending on which group) from our clinic and should be replaced at least bimonthly to:
  1. compensate subjects for their time and increase the incentive of the subjects to remain in the study,
  2. encourage regular replacement of contact lens solutions, and
  3. encourage subjects to return for bimonthly visit.

***Confidentiality***

Only members in this project are allowed to have access to the collected information. Results from the current study may be published but no personal information will be disclosed.

***Subject number***

From the current ROMIO study, the mean different and SD of the change in axial length in the ortho-k and the control groups who have completed the 12-month lens wear timeline are 0.12 and 0.17mm respectively. To detect a 0.12mm (equivalent to 0.33D) between-group difference for an alpha value of 0.05 and 80% power in 12 months, a sample size of 33 is required for each group.

For Experiment 1, we aim to have at least 66 ortho-k and 33 control subjects completing this study. Allowing for about 15% dropout, we will recruit 80 ortho-k and 40 control children.

*The ROMIO, HM-PRO and TO-SEE studies are ortho-k projects currently being conducted in PolyU. ROMIO and HM-PRO studies are targeting 30 ortho-k and 30 control subjects each and TO-SEE study is targeting for 30 ortho-k subjects. Hence, there will be 90 potential ortho-k and 60 control subjects and so we do not expect difficulties in recruiting the required subjects for this experiment*.

For Experiment 2, we aim to have at least 70 (35 younger and 35 older) subjects completing the study. Allowing for dropouts (including unsuccessful lens wear), we will recruit 90 (45 younger and 45 older) children.

***Study period***

Experiments 1 and 2 can commence at the same time. For Experiment 1, each subject will be followed for 14 months. Subjects in the 2-year myopic control studies will be returning for their final visit in the next 12 months. Assuming that the subjects required in Experiment 1 can be recruited in 12 months and 2 months to complete data collection, the study period will be at least 28 months (12+14+2).

For Experiment 2, each subject will be followed for 13 months. Assuming 6 months for subject recruitment, and 2 months for data collection, the study period will be 22 months (6+14+2). As the two experiments can be conducted concurrently, and allowing for delays (eg delays in recruitment or subject returning for data collection), the estimated study period will be at least 28 months (Table 5).

***Manpower***

We will require at least two examiners. A masked examiner for the cycloplegic examination at the data collection visits and an unmasked examiner for ortho-k fitting, i.e. two full time research staff (one project fellow and one research assistant (RA)*) will be needed. We will need an administrative staff to help with booking of patients, lens ordering, record keeping and data entries etc. Both research personnel must be experienced ortho-k practitioners.

(*Will convert this post to a MPhil position if the RA is interested in further study)

***Outputs***

At least two papers and three conference presentations

**Table 1. Data collection visits for Experiments 1 & 2.**

| Cycloplegic | Non-cycloplegic | Description | Remark |
| --- | --- | --- | --- |
| Phase I |  |  |  |
| I-0 |  | Baseline |  |
| [RS period: Weekly visits (for Group OKd subjects) to determine stabilization of Rx. Order new ortho-k/spectacles for the 3 groups of subjects for delivery at Visit I-1 (Rx for Group C and OKc should not be more than 1 month old)] | | | |
| I-1 |  | End of RS period in Phase I (±3 days)  (*same visit as I-0 for Experiment 2 as there will be no RS period) | - Delivery of glasses for Group C and ortho-k lenses for Group OKc - Delivery of glasses for Group OKd and all subjects in Experiment 2 |
|  | I-3 | 3 (±1 week) after commencing Phase I |  |
|  | I-6 | 6 months (±1 week) after commencing Phase I | - Order new ortho-k lenses/spectacles based on updated Rx for the 3 groups of subjects for Visit I-7 where indicated |
| I-7 |  | 7 months (±1 week) after commencing Phase I |  |
| Phase II |  |  |  |
| II-0 |  | Equivalent to Visit I-7 | - Glasses for Group C and ortho-k lenses for Group OKc (if deemed necessary) - Ortho-k lenses for Group OKd and all subjects in Experiment 2 |
| II-1 |  | End of RS period (±3 days) |  |
|  | II-3 | 3 (±1 week) after commencing Phase II |  |
|  | II-6 | 6 months (±1 week) after commencing Phase II |  |
| II-7 |  | 7 months (±1 week) after commencing Phase II |  |

RS - Rx-Stabilization

Group C – Control group in Experiment 1

Group OKc – Ortho-k subjects who will continue ortho-k lens wear for the whole experiment period in Experiment 1

Group OKd – Ortho-k subjects who will discontinue ortho-k lens wear in Phase I and resume ortho-k lens wear in Phase II in Experiment 1

**Table 2. Contact lens solutions and lens accessories used.**

| **Products** | **Brand** | **Supply per 7 months per subject** |
| --- | --- | --- |
| Disinfecting solution | Menicon Menicare Plus | 4 bottles |
| Daily cleaner | Menicon O2 Care | 4 bottles |
| Saline | AMO LensPlus Purite | 7 bottles |
| Enzymatic cleaner | Menicon Progent | 4 boxes |
| Artificial tears | Alcon Tears Naturale Free | 14 boxes |
| Lens cases | AMO lens cases | 7 pieces |

**Table 3. Inclusion criteria for new orthokeratology lens wear**

| Age | - 6-15 years of age on the date of recruitment |
| --- | --- |
| Refractive error Myopia  Cylinder | - Non-cycloplegic manifest refraction - ≥ 1.50DS and ≤ 4.50DS in manifest refraction - ≤ 3.00DC for axis 180±30 or ≤ 1.00DC for other axes |
| Visual acuity | - Best corrected monocular logMAR visual acuity 0.10 or better |
| Ocular health | - Eyes straight at distance and near with best subjective correction - No abnormal ocular health - No ocular conditions which might affect vision or vision development (for example, cataract and ptosis) - No previous rigid contact lenses (including orthokeratology lenses) experience - No contraindications for overnight orthokeratology lens wear. |
| General health | - No systemic conditions which might affect the vision or vision development |
| Others | - No previous experience in myopic treatment (e.g. refractive surgery or progressive lens wear for myopic control) - Willing to wear orthokeratology lenses in accordance with the instructions from practitioner - Willing to follow up the protocol of the study |

**Table 4a. Examination procedures for all subjects**

|  | **Data collection** | | **Ortho-k aftercare** | **Visits in RS period** |
| --- | --- | --- | --- | --- |
|  | **Cycloplegic** | **Non-cycloplegic** |  |  |
| Ortho-k subjects | **✓** | **✓** | **✓** | **✓** |
| Control subjects | **✓** | **✓** | **--** | **--** |
| **Procedures** |  |  |  |  |
| 1. Habitual/Unaided VA |  |  |  |  |
| high contrast | UM | UM | UM | UM |
| low contrast | UM | UM | UM | UM |
| 2a. Subj  2b. Obj refraction | UM | UM | UM | UM |
| 3. Best corrected VA |  |  |  |  |
| high contrast | UM | UM | UM | UM |
| low contrast | UM |  |  |  |
| 4. Photo-biomicroscopy | UM | UM | UM | UM |
| 5. Medmont | UM | UM | UM | UM |
| 6. ORA | UM | UM |  |  |
| 7. Pentacam | UM | UM |  |  |
| 8. SP-2000P/ConfoScan 4 | UM |  |  |  |
| 9. Non-cyclo IOLMaster^TM^ | M | M | M | M |
| 10. Cyclo IOLMaster^TM^ | M |  |  |  |
| 11. Cyclo COAS | UM |  |  |  |
| 12a. Cyclo subj  12b. Obj refraction  12c. Peripheral Refraction | UM |  |  |  |
| 13. Cyclo Medmont | UM |  |  |  |
| 14. Cyclo Pentacam | UM |  |  |  |

UM: unmasked M: masked

**Table 4b. Instrumentation**

| **Procedures** | **Instrument** |
| --- | --- |
| Habitual/Unaided VA | ETDRS charts (Precision Vision, IL, US) 100% contrast |
| Subjective refraction | Retinoscopy, trial frame and trial lenses and cross-cylinder |
| Objective refraction | Shin-Nippon Open Field Autorefractor |
| Best corrected VA | ETDRS charts (Precision Vision, IL, US) |
| Corneal biomechanics | Ocular Response Analyser (ORA) (Reichert Inc, Depew, NY) |
| Noncontact IOP | Nidex auto non-contact tonometer NT4000 |
| Corneal thickness | Pentacam (Oculus, Inc., Lynnwood, WA) |
| Corneal morphology | SP-2000P (Topcon, Tokyo, Japan)  ConfoScan 4 (Nidek, Inc., Freemont, CA) |
| Corneal topography | Pentacam (Oculus, Inc., Lynnwood, WA)  Medmont E300 (Medmont International Pty Ltd, Australia) |
| Photo-biomicroscopy | TOPCON TRC-NW6S and TOPCON IMAGEnet |
| Amplitude of Accommodation | Push-up bar |
| Cycloplegic axial length measurement | Zeiss IOLMaster^TM^ (Zeiss Humphrey, Dublin, CA, USA) |
| Cycloplegic objective refraction | Shin-Nippon Open Field Autorefractor |
| Cycloplegic subjective refraction | Trial frame and trial lenses and cross-cylinder |
| Cycloplegic Aberrometry | Complete Ophthalmic Analysis System (COAS, Wavefront Sciences Ltd, USA) |

**Table 5. Study progress**

| **Months** | **1-4** | **5-8** | **9-12** | | | **13-16** | **17-20** | **21-24** | **25-28** |
| --- | --- | --- | --- | --- | --- | --- | --- | --- | --- |
| **Experiment 1** |  |  |  | | |  |  |  |  |
| * Recruitment |  |  |  | | |  |  |  |  |
| * Monitoring |  |  |  | | |  |  |  |  |
| **Experiment 2** |  |  |  | | |  |  |  |  |
| * Recruitment |  |  |  |  |  |  |  |  |  |
| * Monitoring |  |  |  | | |  |  |  |  |
| * Data collection |  |  |  | | |  |  |  |  |
| **Experiment 3** |  |  |  | | |  |  |  |  |
| * Recruitment |  |  |  | | |  |  |  |  |
| * Monitoring |  |  |  | | |  |  |  |  |
| * Data collection |  |  |  | | |  |  |  |  |

**Supplementary to Table 4a.**

| **Manufacturer of lenses** | **Menicon** | | **Procornea** | |
| --- | --- | --- | --- | --- |
| **Groups** | **Ortho-k** | **Control** | **High Myopes** | |
| **Procedures** |  |  |  |  |
| 1. Habitual/Unaided VA | Unaided | Habitual | Habitual | |
| LCD high contrast | **✓** | **✓** | **✓** | |
| High contrast EDTRS | **✓** | **✓** | **✓** | |
| Low contrast EDTRS | **✓** | **✓** | **✓** | |
| 2a. Subjective Rx | LCD chart + normal room lighting | | | |
| LCD high contrast | **✓** | **✓** | **✓** | |
| 2b. Obj refraction | - Shin-Nippon; Binocular fixation; Auto mode - 3 sets of readings; both sphere and cylinder not exceed 0.25D | | | |
| 3. Best corrected VA | Aided | | | |
| high contrast | **✓** | **✓** | **✓** | |
| low contrast | **✓** | **✓** | **✓** | |
| 4. Photo-biomicroscopy | List of graded items:   - Efron’s scale - Corneal staining: location / coverage / depth - Limbal, conjunctival, papillary injection: location / grade - Conjunctival staining: location / grade - Papillae and follicles: location / grade - Ortho-k related findings: iron ring, white lesion, fribrillary lines (presence/absence; grading) - Photo-documentation   - 16 standard photos for each eye     - Cornea and bulbar conjunctival x 5 gazes (central, inferior, superior, temporal and nasal)       - 5 locations x white light (diffuse light) @ 16X       - 5 locations x blue light (broad beam) @ 16X     - Iron ring (or the absence)       - 1 location x white light (parallelepiped) @ 16X       - 1 location x blue light (broad beam) @ 16X     - Palpebral conjunctiva for upper and lower eyelid       - 2 locations x white light (diffuse light) @ 16X       - 2 locations x blue light (broad beam) @ 16X   - Higher magnification and optic section for special features (appearance and depth) e.g. | | | |

**Supplementary to Table 4a (cont.)**

|  | **Menicon** | | **Procornea** |
| --- | --- | --- | --- |
|  | **Ortho-k** | **Control** | **High Myopes** |
| **Procedures** |  |  |  |
| 5. Medmont | - Cyclo visits: 4 good images per eye - Other visits: two good images per eye | | |
| 6. ORA | - 4 good images per eye:   - image score > 3.6 - perform NCT (TOPCON, 3 readings with difference not exceeding 3 mmHg) only if 3 good results cannot be obtained in 12 trials | | |
| 7. Pentacam | - 25 scans/s; 3 good images per eye | | |
| 8. SP-2000P/ConfoScan 4 | - Central: 3 images; CCT not exceed 10 um - Superior / inferior: 1 image | | |
| 9. Non-cyclo IOLMaster^TM^ | - To be determined | | |
| 10. Cyclo IOLMaster^TM^ | - AL: the first set of 5 readings within 0.02mm - ACD: 3 readings within 0.04mm | | |
| 11. Cyclo COAS | - Monocular fixation; central: 5 readings | | |
| 12a. Cyclo subjective Rx | - LCD chart + normal room lighting | | |
| 12b. Cyclo objective Rx | - Shin-Nippon; Binocular fixation; Auto mode - 3 sets of readings; both sphere and cylinder not exceed 0.25D | | |
| 12c. Peripheral Refraction | - Pupil alignment method - manual mode - quick 5 x 2: 5 out of 10 data will be used   - sphere and cylinder not exceed 0.50D;   - axis not exceed 30 degrees | | |
| 13. Cyclo Medmont | **✓** | **✓** | 🗶 |
|  | - 4 good images per eye | | |
| 14. Cyclo Pentacam | - 25 scans/s; 3 good images per eye | | |

Pertinent data will be determined for both eyes:

* 1-6, 9-10, 12a and 12b

For the other tests (11, 12c, 13-14)

* ROMIO and TO-SEE will continue to perform tests on both eyes

* Only eligible eye will be assessed for HM-PRO
